# Amyloid-β_1-42_ oligomers enhance mGlu_5_R-dependent synaptic weakening via NMDAR activation and complement C5aR1 signaling

**DOI:** 10.1016/j.isci.2023.108412

**Published:** 2023-11-07

**Authors:** Ai Na Ng, Eric W. Salter, John Georgiou, Zuner A. Bortolotto, Graham L. Collingridge

**Affiliations:** 1 School of Physiology, Pharmacology and Neuroscience, University of Bristol, Biomedical Sciences Building, University Walk, Bristol BS8 1TD, UK; 2 Lunenfeld-Tanenbaum Research Institute, Mount Sinai Hospital, Sinai Health System, 600 University Avenue, Toronto, ON M5G 1X5, Canada; 3 Department of Physiology, University of Toronto, Toronto, ON M5S 1A8, Canada; 4 Tanz Centre for Research in Neurodegenerative Diseases, University of Toronto, Krembil Discovery Tower, 60 Leonard Avenue, Toronto, ON M5T 0S8, Canada

**Keywords:** Health sciences, Pathophysiology, Neuroscience, Molecular neuroscience

## Abstract

Synaptic weakening and loss are well-correlated with the pathology of Alzheimer’s disease (AD). Oligomeric amyloid beta (oAβ) is considered a major synaptotoxic trigger for AD. Recent studies have implicated hyperactivation of the complement cascade as the driving force for loss of synapses caused by oAβ. However, the initial synaptic cues that trigger pathological complement activity remain elusive. Here, we examined a form of synaptic long-term depression (LTD) mediated by metabotropic glutamate receptors (mGluRs) that is disrupted in rodent models of AD. Exogenous application of oAβ (1–42) to mouse hippocampal slices enhanced the magnitude of mGlu subtype 5 receptor (mGlu_5_R)-dependent LTD. We found that the enhanced synaptic weakening occurred via both N-methyl-D-aspartate receptors (NMDARs) and complement C5aR1 signaling. Our findings reveal a mechanistic interaction between mGlu_5_R, NMDARs, and the complement system in aberrant synaptic weakening induced by oAβ, which could represent an early trigger of synaptic loss and degeneration in AD.

## Highlights

• Oligomeric amyloid beta (oAβ) enhances hippocampal long-term depression (LTD)
• Enhanced LTD mediated by metabotropic glutamate receptor subtype 5 (mGlu_5_R)
• NMDARs and complement C5aR1 activation essential for oAβ-mediated LTD amplification
• We uncovered a novel signaling axis between glutamatergic receptors and complement

## Graphical abstract

**Figure:**
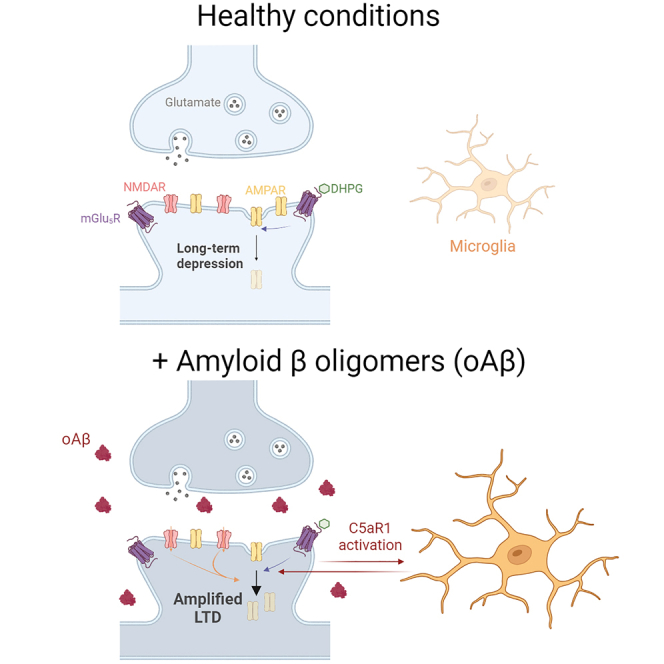

## Introduction

Alzheimer’s disease (AD) is a progressive neurodegenerative disease characterized by memory loss, cognitive deficits, and changes to personality and behavior.^1^ Although it is the most common cause of dementia affecting the aging population worldwide, therapeutic options are limited. Weakening and subsequent loss of synapses are early events in AD progression that precede the deterioration of memory and cognitive functions.^2^^,^^3^^,^^4^ The development of improved treatment options requires a deeper understanding of how synapses are initially impaired and how this leads to overt neuronal degeneration.

A key pathological feature of AD is plaques, which are made up primarily of insoluble aggregates of amyloid beta (Aβ) protein derived from the amyloid precursor protein (APP). The original amyloid cascade hypothesis posited that these Aβ plaques are the initial trigger of AD pathology.^5^ However, subsequent research found that levels of soluble oligomers of Aβ (oAβ) better correlate with the degree of cognitive impairment in AD patients.^6^^,^^7^ Subsequently, oAβ has been found to bind excitatory synapses leading to disruption of synaptic function.^8^^,^^9^^,^^10^ Normally, synaptic strength is modulated through long-term potentiation (LTP) and long-term depression (LTD), which are thought to provide the cellular and molecular basis of learning and memory.^11^ oAβ mediates a shift in the LTP/LTD balance, favoring LTD over LTP, leading to eventual net synapse loss.^3^^,^^12^^,^^13^^,^^14^^,^^15^^,^^16^^,^^17^^,^^18^^,^^19^^,^^20^ Therefore, the study of oAβ-dependent changes to LTD provides a means to investigate the initial synaptic changes relevant to the earliest stages of AD.

Synapse damage and loss caused by oAβ does not occur in a neuron-autonomous manner. The complement cascade is an innate immune pathway which has emerged as a central driver of synapse loss in AD.^21^^,^^22^ Complement cascade activation converges on the cleavage of complement component 3 (C3), generating C3a and C3b, the latter of which covalently attaches to target structures.^23^ Subsequently, C3b can either be degraded to iC3b to mediate phagocytosis via the CR3 receptor expressed on phagocytes or can form the C5 convertase along with C4b and C2a. Analogous to C3, the C5 convertase cleaves C5 to generate the fragments C5a and C5b. C5a is a diffusible protein fragment that promotes chemotaxis and inflammation primarily through binding to the receptor C5aR1. Upregulation of C5aR1 surrounding plaques in AD mouse models has been observed.^24^ Further, blockade of C5aR1 signaling using either pharmacological or genetic inhibition has provided convergent evidence for the causative role of C5aR1 in driving tissue pathology and behavioral impairments in multiple AD mouse models.^25^^,^^26^^,^^27^^,^^28^ However, the mechanism by which C5aR1 signaling generates neuronal damage in AD is poorly understood.

LTP and LTD of glutamatergic synapses are mediated by activation of N-methyl-D-aspartate receptors (NMDARs) and/or metabotropic glutamate receptors (mGluRs).^29^^,^^30^^,^^31^ LTD, induced either by NMDARs or mGluRs, can trigger subsequent synapse elimination.^32^^,^^33^^,^^34^^,^^35^ Both mGluR- and NMDAR-dependent signaling are affected by oAβ, leading to synaptic plasticity impairments and aberrant synapse loss.^3^^,^^13^^,^^14^^,^^15^^,^^17^^,^^36^^,^^37^^,^^38^^,^^39^

Synaptic plasticity involving the mGluR subtype 5 (mGlu_5_R) is particularly pertinent to the understanding of AD for multiple reasons. For example, mGlu_5_R was found to be the only co-receptor of cellular prion protein (PrP_c_) necessary for oAβ to activate intracellular signaling in neurons.^40^ Further, both knockout of mGlu_5_R and pharmacological antagonism is protective in oAβ-based and genetic models of AD.^39^^,^^41^^,^^42^^,^^43^ Interestingly, the complement cascade has recently been implicated in oAβ-dependent synapse loss mediated by mGluR activation.^44^ A silent allosteric modulator of mGlu_5_R has also been found to restore synapse density through reduced tagging by C1q and microglia engulfment in a genetic mouse model of AD.^45^

Previously, we identified a simple method to study mGluR-mediated LTD, via the brief application of the group I mGluR agonist S-3,5-dihydroxyphenylglycine (DHPG), which causes lasting depression of AMPAR-mediated synaptic transmission.^46^ This DHPG-induced LTD (DHPG-LTD) has since been used extensively to uncover many aspects of mGluR physiological and pathological function, including the regulation of mGluR function in AD mouse models.^13^^,^^15^^,^^16^^,^^36^^,^^47^^,^^48^ Here, we have used DHPG-LTD to investigate whether C5aR1 signaling is involved in mGluR-driven synaptic plasticity. We applied oAβ as a standard way to induce synaptotoxicity that is relevant to AD pathology. We found that oAβ enhanced DHPG-LTD via a mechanism that involves the activation of mGlu_5_Rs, NMDARs and C5aR1 of the complement cascade. These findings reveal a signaling axis between glutamate receptors and the complement cascade that triggers early synaptic dysfunction that may underlie the initial stages of dementia.

## Results

### Acute oAβ exposure enhances mGlu_5_R-dependent LTD

To investigate the impact of oAβ on synaptic function, we incubated acute hippocampus slices for 2 h with oAβ (500 nM). We subsequently measured excitatory synaptic transmission and plasticity with field potential recordings at hippocampal CA3-CA1 synapses. As a positive control for the bioactivity of our oAβ preparation, we confirmed previous findings^15^^,^^49^ that oAβ was able to impair LTP in slices pretreated with oAβ (+oAβ) versus interleaved non-treated slices (-oAβ; Figures S1A and S1B). Although LTP was impaired by acute application of oAβ, basal synaptic transmission was unaltered, indicated by the overlapping input-output fEPSP relationship in +oAβ compared to -oAβ slices (Figures S1C and S1D). As such, this oAβ application paradigm allowed us to study early disruptions to plasticity that precedes the loss of synapses.

**Figure 1:**
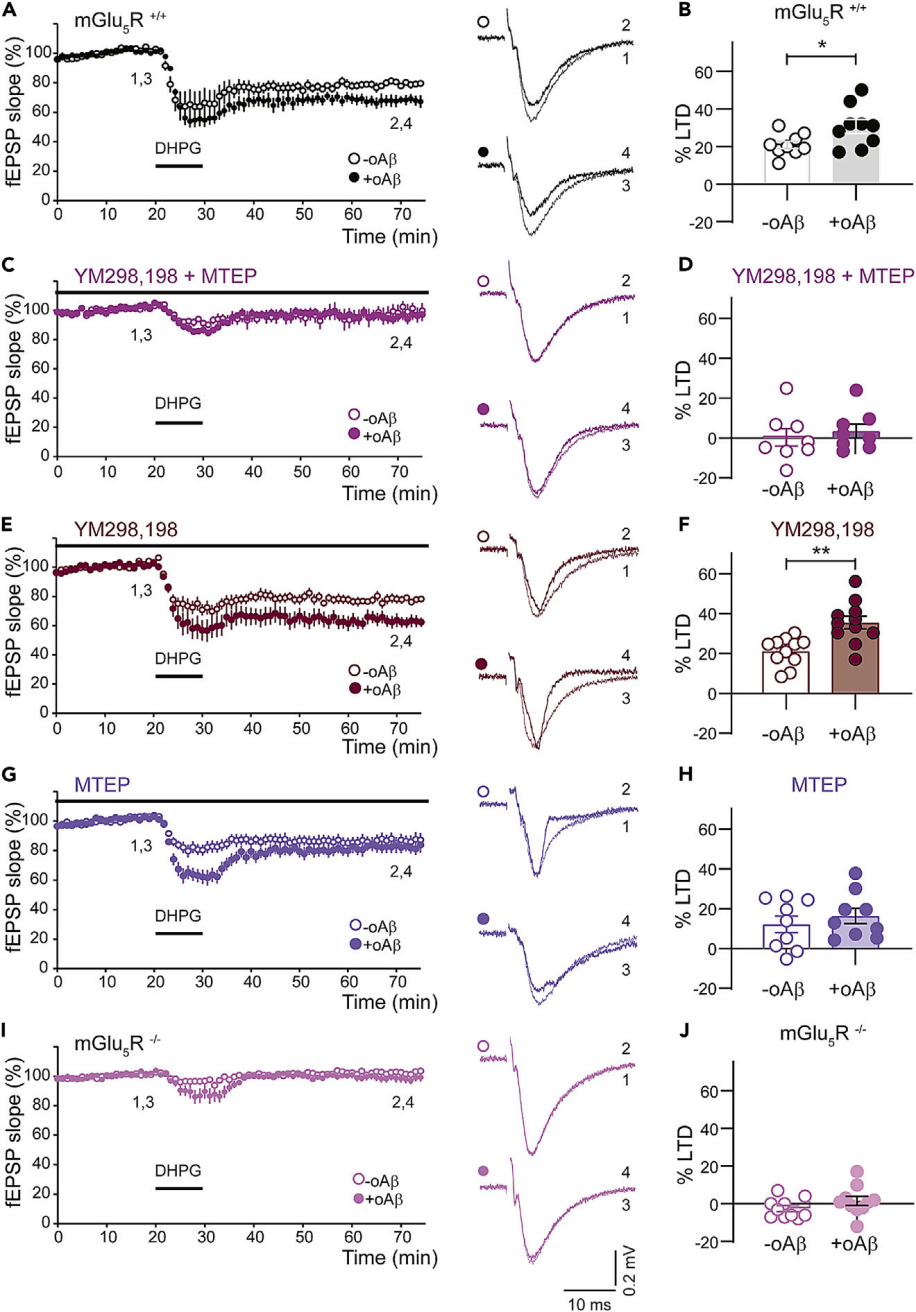
oAβ enhances mGlu_5_R-LTD at CA3-CA1 excitatory synapses (A and B) Application of *S*-DHPG (50 μM, 10 min), induced LTD in -oAβ slices from mGlu_5_R^+/+^ mice (21 ± 2%; n = 9). Interleaved +oAβ slices showed an enhancement of DHPG-LTD (31 ± 4%; n = 9; p = 0.032 [∗]). DHPG-LTD magnitude is quantified in B. (C and D) Combined application of mGlu_1_R and mGlu_5_R antagonists YM298,198 (2 μM) and MTEP (1 μM), respectively, blocked the induction of DHPG-induced LTD in both -oAβ and +oAβ conditions. Quantification of LTD magnitude is shown in D. There was no significant difference between -oAβ (0 ± 5%; n = 8) and +oAβ treated slices (4 ± 4%; n = 8). (E and F) Enhancement of DHPG-LTD by oAβ in the presence of YM298,198 (2 μM) alone. Quantification of LTD magnitude shown in F. LTD magnitude was significantly higher in oAβ-treated slices (37 ± 4%; n = 11) compared to -oAβ controls (22 ± 2%; n = 11; p = 0.0012 [∗∗]). (G and H) In the presence of MTEP (1 μM) alone, DHPG-LTD was not significantly different between -oAβ (13 ± 4%; n = 9) and +oAβ-treated slices (18 ± 4%; n = 9). LTD magnitude quantification is shown in H. (I and J) In slices from mGlu_5_R^−/−^ mice, DHPG failed to induce LTD in either condition (+oAβ = 2 ± 3%, n = 10; -oAβ = −3 ± 2%, n = 10). Quantification of LTD magnitude is shown in J. There was no significant difference between -oAβ and +oAβ treated slices. Sample fEPSP traces in all panels are the mean of 4 consecutive responses at the indicated time points (1–4). For each experiment, sample traces before and after DHPG treatment are superimposed. Two-tailed t tests were used in B, D, F, H, and J. In all graphs, data are presented as mean ± SEM.

To investigate how oAβ alters mGluR-dependent synaptic plasticity mechanisms, we induced LTD using *S*-DHPG (referred to hereafter as DHPG), a selective agonist for group I mGluRs (mGlu_1_R and mGlu_5_R), that is widely used to study mGluR function. A short application of DHPG (10 min) induced a stable LTD (DHPG-LTD) in -oAβ slices from wild-type mice (Figures 1A and 1B). We observed that the magnitude of DHPG-LTD was significantly enhanced in interleaved +oAβ slices (Figures 1A and 1B). We then sought to determine the group I mGluR subtype responsible for mediating the enhancement of DHPG-LTD by oAβ. We co-applied the mGlu_1_R and mGlu_5_R antagonists, YM298,198 (2 μM) and MTEP (1 μM), respectively. Under these conditions, the induction of DHPG-LTD was blocked in both -oAβ and +oAβ slices (Figures 1C and 1D), indicating that both control and oAβ-enhanced DHPG-LTD shared a group I mGluR-dependency. Next, to determine whether the LTD phenotype exhibited a group I mGluR subtype-specificity, we applied each antagonist independently and measured DHPG-LTD in the presence and absence of oAβ. YM298,198 failed to prevent the enhancement of DHPG-LTD by oAβ, indicated by the significantly greater LTD magnitude in +oAβ compared to -oAβ slices (Figures 1E and 1F). Conversely, in the presence of MTEP, the level of DHPG-LTD was not significantly different between control and oAβ-treated slices (Figures 1G and 1H). This suggested that specifically the mGlu_5_R subtype was responsible for mediating the effects of oAβ on DHPG-LTD. To examine this using an orthogonal approach to interfere with mGlu_5_R activity, we examined DHPG-LTD in mice genetically lacking mGlu_5_R.^50^ We observed that in mGlu_5_R^−/−^ mice, DHPG-LTD was absent in both -oAβ and +oAβ slices (Figures 1I and 1J). Together, our pharmacological and genetic data indicate that the enhancement of DHPG-LTD by oAβ requires the activation specifically of mGlu_5_Rs (referred to hereafter as mGlu_5_R-LTD).

### NMDARs are required for oAβ-mediated enhancement of mGlu_5_R-LTD

**Figure 2:**
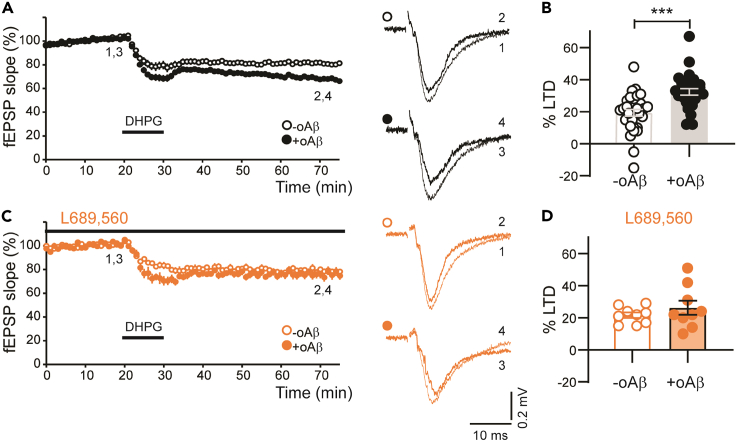
Enhancement of mGlu_5_R-LTD by oAβ requires NMDARs (A and B) In a separate cohort, DHPG-LTD was enhanced in slices treated with oAβ (34 ± 2%, n = 22) compared to the interleaved -oAβ slices (21 ± 2%, n = 22; p = 0.00031 [∗∗∗]). Quantification of LTD magnitude shown in B. (C and D) In the presence of the NMDAR antagonist L689,560 (5 μM), the magnitude of DHPG-LTD was not significantly different between +oAβ slices (26 ± 4%, n = 9) and -oAβ slices (22 ± 2%, n = 9). Quantification of LTD magnitude shown in D. Sample fEPSP traces in all panels are the mean of 4 consecutive responses at the indicated time points (1–4). For each experiment, sample traces before and after DHPG treatment are superimposed. A two-tailed t test was used in B and D. In all graphs, data are presented as mean ± SEM.

There are two distinct forms of DHPG-LTD that can be distinguished by their dependence upon the activation of NMDARs.^46^^,^^51^ To determine whether NMDARs are required for mGlu_5_R-LTD either under control conditions or the enhanced mGlu_5_R-LTD observed in the presence of oAβ, we used a selective NMDAR antagonist, L689,560 (5 μM). L689,560 was chosen as it acts at the glycine site of the NMDAR and so is independent of L-glutamate concentration, which might be altered by oAβ treatment.^16^ In these experiments, we interleaved a new set of controls and again observed greater mGlu_5_R-LTD magnitude in +oAβ compared to -oAβ slices (Figures 2A and 2B). We found that NMDAR activation was not required for mGlu_5_R-LTD in control (-oAβ) conditions. However, the enhancement of mGlu_5_R-LTD by oAβ was completely prevented by L689,560 (Figures 2C and 2D) and therefore requires NMDAR signaling.

### oAβ enhances mGlu_5_R-LTD via C5aR1 signaling

Early studies on the effects of oAβ on synaptic plasticity found that inhibition of LTP was prevented by application of minocycline, an inhibitor of microglia activation.^52^ Further, it has recently been found that agonism of group I mGluRs *in vivo* leads to complement cascade activation and eventual synapse loss.^44^ We, therefore, hypothesized that complement cascade activity is necessary for the enhancement of mGlu_5_R-LTD by oAβ. Within the complement cascade, we chose to examine the role of C5aR1 signaling, which has been implicated in the pathology of genetic mouse models of AD.^25^^,^^26^^,^^27^^,^^28^^,^^53^^,^^54^

**Figure 3:**
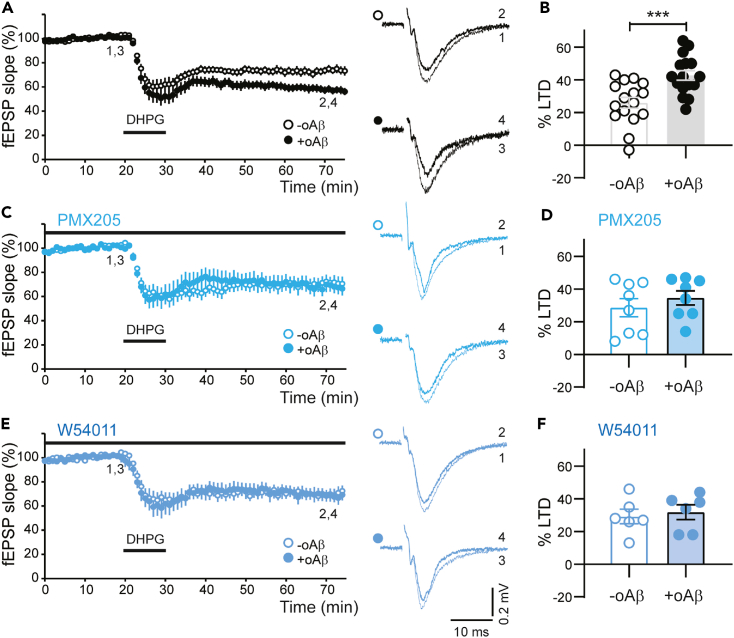
Complement C5aR1 is necessary for oAβ-driven enhancement of mGlu_5_R-LTD (A and B) In a third cohort, oAβ-treated slices had significantly greater DHPG-LTD magnitude (43 ± 3%, n = 16) compared to the interleaved -oAβ slices (26 ± 3%, n = 16; p = 0.00064 [∗∗∗]). Quantification of LTD magnitude shown in B. (C and D) In the presence of the C5aR1 antagonist PMX205 (0.3 μM), the level of DHPG-LTD was not significantly different between +oAβ slices (35 ± 4%, n = 8) and -oAβ slices (29 ± 6%, n = 8). Quantification of LTD magnitude shown in D. (E and F) A separate C5aR1 antagonist, W54011 (0.3 μM), also prevented the enhancement of DHPG-LTD magnitude in +oAβ slices (32 ± 5%, n = 6) compared to -oAβ slices (29 ± 4%, n = 6). Quantification of LTD magnitude shown in F. Sample fEPSP traces in all panels are the mean of 4 consecutive responses at the indicated time points. For each experiment, sample traces before and after DHPG treatment are superimposed. Two-tailed t tests were used in B, D, and F. In all graphs, data are presented as mean ± SEM.

For these experiments, we also interleaved a new set of controls and again observed that mGlu_5_R-LTD magnitude was significantly higher in +oAβ compared to -oAβ slices (Figures 3A and 3B). To investigate the role of C5aR1 in this phenotype, we bath-applied PMX205 (0.3 μM), a peptide-based C5aR1 antagonist.^55^ In control (-oAβ) slices, DHPG application still induced a stable LTD in the presence of PMX205. However, there was no significant difference in mGlu_5_R-LTD magnitude between -oAβ and +oAβ slices (Figures 3C and 3D). These data suggested that C5aR1 signaling had a specific role only in the oAβ-enhanced portion of mGlu_5_R-LTD. To provide further evidence for this, we also used a non-peptide based C5aR1 antagonist, W54011.^56^ As with PMX205, bath-application of W54011 (0.3 μM) had no effect on the level of mGlu_5_R-LTD induced under control conditions (-oAβ) but prevented the enhancement induced by oAβ (Figures 3E and 3F). Together, these data indicate that the oAβ-mediated enhancement of mGlu_5_R-LTD at hippocampal CA3-CA1 synapses occurs via activation of complement C5aR1 signaling.

## Discussion

The complement cascade is emerging as a key innate immune pathway for shaping synapses during brain development, as well as driving synapse loss in various disease states.^22^^,^^57^^,^^58^ However, the synaptic signals that lead to complement activation remain poorly understood. In the present study, we uncover a mechanistic link between innate immune signaling and the amplification of glutamatergic synapse depression induced by oAβ. We found that mGlu_5_R-dependent LTD induced by DHPG application was enhanced in slices treated with oAβ (Figure 1). Unlike vehicle-treated slices, the oAβ-mediated enhancement of mGlu_5_R-LTD was dependent on NMDAR signaling (Figure 2). Similarly, antagonism of the complement cascade receptor C5aR1 prevented oAβ from enhancing mGlu_5_R-LTD (Figure 3), without affecting the underlying mGlu_5_R-LTD *per se*. Given that basal synaptic transmission was unaltered by oAβ (Figure S1), our findings reveal a signaling axis between mGlu_5_Rs, NMDARs, and the complement cascade that mediates early synaptic plasticity alterations, upstream of overt synapse loss.

### oAβ-induced glutamatergic synapse dysfunction

Although AD is an extremely slowly progressing disease, it is likely that at the level of a single synapse there is acute damage, which in many cases may be orchestrated by toxic oAβ species. This would presumably occur when the processes that ordinarily prevent the accumulation of oAβ are sufficiently compromised such that the local concentration increases to a level that can impair synaptic function and structure. Therefore, the transient application of oAβ has been widely employed to model this early, critical stage of the disease. Consistent with the validity of this model, mechanisms that are engaged by the acute application of oAβ to impact synaptic plasticity, such as the involvement of GSK-3, caspase-3,^17^ tau,^19^^,^^59^ and microglia^52^ are all directly relevant to human AD pathology.

Multiple studies have found that oAβ causes a shift in the LTP-LTD balance, favoring LTD.^13^^,^^14^^,^^15^^,^^16^^,^^17^^,^^36^^,^^49^^,^^60^^,^^61^ Altered activity of both group I mGluRs and NMDARs by oAβ is central to this glutamatergic synaptic failure. mGlu_5_R was identified as a co-receptor of cellular prion protein (PrP_c_) for binding oAβ and is necessary for oAβ-induced intracellular signal transduction and synapse loss.^37^^,^^40^^,^^62^^,^^63^ Additionally, oAβ binds to NMDARs^64^^,^^65^ and impairs NMDAR-dependent LTP while enhancing NMDAR-dependent LTD.^13^^,^^14^^,^^15^^,^^17^^,^^19^^,^^39^^,^^52^^,^^60^^,^^61^^,^^66^

Despite these important advances, whether NMDARs and mGluRs are synergistically involved in oAβ-induced synaptic weakening and loss is poorly understood. Of relevance to the present study, at least two distinct forms of DHPG-LTD exist, which can be distinguished by the dependence on NMDARs.^46^^,^^51^ In our current study, DHPG-LTD induction under control conditions was not affected by NMDAR antagonism and was completely dependent on group I mGluRs. The finding that full inhibition of DHPG-LTD pharmacologically required antagonism of both mGlu_1_Rs and mGlu_5_Rs but was also absent in mGlu_5_R ^−/−^ mice alone, is most readily explained by the receptor being an mGlu_1_/mGlu_5_ heterodimer.^67^^,^^68^ In mGlu_5_R^−/−^ mice, these mGlu_1_/mGlu_5_ heterodimers would fail to assemble thereby completely preventing the induction of DHPG-LTD. In contrast, the oAβ-enhanced portion of the LTD fully depended on the activation of NMDARs and specifically required mGlu_5_Rs but not mGlu_1_Rs. Therefore, oAβ may preferentially induce and/or bias toward mGlu_5_R-dependent signaling, which is blocked by MTEP but not YM298,198.

Our observations of enhanced mGlu_5_R-LTD in the presence of exogenously applied oAβ are in line with previous studies using acute oAβ exposure.^15^^,^^16^^,^^47^ Conversely, studies utilizing amyloidogenic AD models with overexpression of APP have observed an inhibition of mGluR-LTD.^13^^,^^48^ One explanation for these disparate findings is the duration of oAβ exposure between AD models. In models of acute oAβ exposure (including this study), few if any synapses would be expected to undergo endogenous mGlu_5_R-LTD in the presence of oAβ prior to experimental LTD induction utilizing DHPG. Conversely, in models utilizing APP overexpression, oAβ exposure is chronic, providing a longer time window for endogenous mGlu_5_R-LTD to occur in the presence of oAβ. Thus, the impairment of mGlu_5_R-LTD in these models may be the result of occlusion. A non-mutually exclusive alternative explanation is that synaptotoxic effects of oAβ require accumulation in intracellular compartments.^69^^,^^70^ In such a scenario, exogenously applied oAβ would need to enter neurons via uptake by surface receptors which could include mGlu_5_R and/or PrP_c_, a known binding partner of mGlu_5_R and oAβ. Conversely, in AD models using overexpression, oAβ is already present intracellularly and would not require an active uptake mechanism.

Both mGlu_5_R and NMDARs are also implicated in AD pathogenesis in genetic mouse models and humans. Antagonists of mGlu_5_R and genetic deletion have both been found to reduce synaptic and behavioral deficits in AD mouse models.^39^^,^^41^^,^^42^^,^^45^^,^^71^ Indeed, the mGlu_5_R silent allosteric modulator BMS-984923,^71^ is currently in phase I clinical trials. Evidence that altered NMDAR signaling is causally related to the human condition is the beneficial action of memantine, a non-competitive NMDAR antagonist, for the treatment of AD. Memantine slows the progression of dementia and its underlying therapeutic mechanism has been attributed to the normalization of synaptic plasticity.^72^^,^^73^^,^^74^ Our experiments reveal a mechanism by which NMDARs and mGlu_5_Rs may act synergistically to control synaptic integrity.

### C5aR1 signaling in AD

In both postmortem human samples as well as numerous AD mouse models, many components of the complement cascade have been found to be upregulated.^22^^,^^58^ Importantly, pharmacological and genetic manipulations have provided evidence that C5aR1 signaling is necessary for synaptic dysfunction and cognitive impairments in AD genetic mouse models. In particular, one of the C5aR1 antagonists used in the current study, PMX205, has been found to reduce pathology in genetic mouse models of AD.^26^^,^^27^ Furthermore, C5aR1 genetic ablation is protective in AD mouse models.^25^^,^^28^ However, the role of oAβ in detrimental C5aR1 signaling, and its intersection with glutamatergic synaptic signaling to cause this dysfunction was previously not understood. Our study provides insight into this communication, by demonstrating that oAβ enhances synaptic weakening, via an NMDAR and mGlu_5_R synergistic mechanism that requires C5aR1.

An important avenue for future studies to investigate is the mechanism by which C5aR1 activation leads to increased LTD. Transcriptomic data indicate that C5aR1 expression is restricted to microglia in the brain^75^ and upregulated C5aR1 protein surrounding Aβ plaques has been found to co-localize with microglia.^24^ Thus, it is likely that a paracrine signal would need to be released by microglia to act on synapses to translate C5aR1 activation into synapse strength changes. The canonical inflammatory function of C5a-C5aR1 includes cytokine production.^23^^,^^76^ Interestingly, neurons are known to express cytokine receptors, the activation of which can induce synaptic plasticity.^77^^,^^78^ Additionally, activation of a different microglia receptor, CR3, during hypoxic and neuroinflammatory conditions leads to the production of reactive oxygen species, which acted in a paracrine fashion to induce AMPAR endocytosis at synapses.^79^ Therefore, cytokines and/or reactive oxygen species are candidate paracrine signals that could mediate the synaptic effects of C5aR1 activation in the presence of oAβ.

A second interesting area for future work is to determine whether the enhanced mGlu_5_R-LTD in the presence of oAβ is associated with differential structural outcomes compared to physiological mGlu_5_R-LTD. C3b, generated by the cleavage of C3, can either form part of the C5 convertase or can be degraded to iC3b, which induces phagocytosis upon binding to its receptor CR3. *In vivo* oAβ administration has been found to induce synapse elimination by microglia via CR3.^80^ Further, complement-dependent synapse pruning by microglia under physiological conditions has been found to be preferentially targeted toward weaker synapses.^81^^,^^82^ Therefore, an intriguing possibility is that upon oAβ exposure, the NMDAR- and C5aR1-mediated enhancement of mGlu_5_R-LTD identified in this study serves as the trigger for subsequent elimination of synapses by microglia.

### Relevance to other neurodegenerative diseases

Pathological upregulation of complement cascade proteins and activity is not exclusive to AD.^83^ For example, studies from *in vitro* and mouse models of amyotrophic lateral sclerosis (ALS) have also identified the role for C5a/C5aR1 in driving pathology.^84^^,^^85^ Interestingly, mGlu_5_R has separately been identified as an emerging therapeutic target in ALS as well.^86^^,^^87^ Further, activation of upstream complement cascade components as well as aberrant mGlu_5_R signaling have been identified as central to the pathology of Huntington’s disease, Parkinson’s disease, and multiple sclerosis.^88^^,^^89^^,^^90^^,^^91^^,^^92^^,^^93^^,^^94^^,^^95^^,^^96^^,^^97^^,^^98^^,^^99^^,^^100^^,^^101^ Therefore, it will be critical for future studies to elucidate whether the pathological mGlu_5_R-NMDAR-C5aR1 signaling axis identified in our study underlies synapse deterioration in multiple brain disorders.

### Limitations of the study

Our chosen experimental paradigm (acute oAβ application to brain slices) permitted the precise control of the exposure timing and species of Aβ. However, this paradigm does not capture chronic aspects of AD or non-amyloidogenic signaling pathways. Therefore, it will be essential for future work to determine whether the mGlu_5_R-NMDAR-C5aR1 axis identified in our study plays a key pathogenic role in various genetic mouse models of AD. Further, experiments in such mouse models will permit the investigation of whether enhanced mGlu_5_R-mediated LTD through NMDAR/C5aR1 activation has a causal role in behavioral impairments observed in AD mouse models.

### Concluding remarks

mGluRs, NMDARs, and C5aR1 have been independently studied for their role in mediating the deleterious effects of oAβ. Our study has found a direct link which places glutamate receptor-innate immune interactions at the center of early synaptic plasticity dysfunction. As oAβ is thought to be a key trigger in AD, a deeper understanding of the cellular and molecular mechanisms of these interactions has the potential to elucidate therapeutic targets which halt synaptic dysfunction and AD progression.

## STAR★Methods

### Key resources table

**Table:** 

REAGENT or RESOURCE	SOURCE	IDENTIFIER
**Chemicals, peptides, and recombinant proteins**		

Aβ(1-42) peptide	MilliporeSigma	Cat. # AG912
1,1,1,3,3,3-hexafluoro-2-propanol (HFIP)	MilliporeSigma	Cat. # 105228
(S)-3,5-dihydroxyphenylglycine (DHPG)	Abcam	Cat. # ab120007
YM298,198	Abcam	Cat. # ab120015
3-((2-Methyl-4-thiazolyl)ethynyl)pyridine (MTEP)	Tocris	Cat. # 2921
PMX205	Tocris	Cat. # 5196/1
W54011	Tocris	Cat. # 5455/10
L689,560	Tocris	Cat. # 0742

**Experimental models: Organisms/strains**		

Mouse: C57BL/6JOlaHsd	Envigo	N/A
Mouse: B6;129-*Grm5tm1Rod*/J	Lu et al., 1997	RRID:IMSR_JAX:00 3121

**Software and algorithms**		

WinLTP	Anderson & Collingridge, 2007	www.WinLTP.com

**Other**		

Slicemate recording chamber	Scientifica	N/A
McIlwain tissue chopper	Ted Pella	N/A
Platinum-iridium bipolar electrode	FHC	N/A
DS2 constant voltage isolator	Digitimer	N/A
Multiclamp 700B amplifier	Molecular Devices	N/A

### Resource availability

#### Lead contact

Further information and requests for resources related to this study should be directed to, and will be fulfilled by, the lead contact Graham Collingridge (collingridge@lunenfeld.ca).

#### Materials availability

This study did not generate new unique reagents.

#### Data and code availability

• All data reported in this paper will be shared by the lead contact upon reasonable request
• This paper does not report any original code
• Any additional information required to re-analyze the data reported in this paper is available from the lead contact upon request.

### Experimental model and study participant details

#### Animals

All the animal experiments and procedures were performed in compliance with the UK Animals (Scientific Procedures) Act (1986) and were guided by the Home Office LiaisonTeam at the University of Bristol. All animal procedures relating to this study were approved by the Animal Welfare and Ethics Review Board at the University of Bristol (approval number UIN/18/059). Ten - 16-week old C57BL/6JOlaHsd male mice from Envigo (Bicester, U.K.) and a mGlu_5_R knockout line,^50^ backcrossed more than 10 generations to a C57BL/6J background, were used in this study. We chose to use male mice due to the recently reported sex specificity of oAβ-mGlu_5_R binding which is present only in males.^39^ Animals were housed four per cage in a room with designated 12 h light/dark cycle (lights on at 08:15/ off at 20:15) with temperature at 21 ± 2°C and access to food and water *ad libitum.* Genomic DNA from mGlu_5_R knockout litters were isolated from tail tips with DNeasy blood and tissue kit (Qiagen; Hilden, Germany; cat. no. 69504) and amplified using polymerase chain reaction. The amplified products were resolved and visualized in ethidium bromide-stained agarose gel. The following primers were used for genotyping:

Common: 5’-CCA TGG CTC GGG CTT GCT GGG CAT C-3’

WT: 5’-GGT GGT GGC TCA CAT GCC AGG TGA C-3'

KO: 5’-GGG GAT CGA TCC GTC CTG TAA GTC T-3'

### Method details

#### Oligomeric amyloid-β_1-__42_ (oAβ) preparation

oAβ was prepared following a previously established protocol^102^ as follows: Aβ peptide_(1-42)_ (MilliporeSigma; Burlington, Massachusetts; TFA recombinant human, ultra-pure; cat. no. AG912) was dissolved in 100 % 1,1,1,3,3,3-hexafluoro-2-propanol (HFIP; MilliporeSigma; cat. no. 105228) to a final concentration of 1 mg/mL. The HFIP/peptide mixture was incubated at room temperature for 1 h and sonicated for 10 min in a water-bath sonicator. The mixture was then air-dried under a gentle stream of nitrogen gas for 1.5 h. The peptide crystal was re-suspended in 100 % DMSO, aliquoted to 10 μL per tube (1 mM) and stored at -80°C until experimental use (see next section). We have previously used single molecule, two-colour fluorescence coincidence detection and analysis to estimate the concentration of oligomers in samples prepared using this methodology.^102^ This analysis demonstrated a high proportion of oligomers compared to monomers, and that the oligomers equated to a concentration of 1-5 nM, comprised of ∼60 % 2 - 4 mers, ∼35 % 5 - 10 mers and ∼5 % 11 - 50 mers.

#### Hippocampal slice preparation

Mice were deeply anaesthetized via inhalation with a mixture of 5 % isoflurane / 95 % oxygen and euthanized by cervical dislocation. The brain was rapidly removed (<1 min) and chilled in cold ACSF solution (containing, in mM: 124 NaCl, 3 KCl, 26 NaHCO_3_, 1.25 NaH_2_PO_4_, 10 D-glucose, 2 MgSO_4_ and 2 CaCl_2_; bubbled continuously with 95% O_2_ / 5% CO_2_). Hippocampi were isolated from both hemispheres and transverse slices (400 μm thickness) were prepared from the dorsal ends of both hippocampi using a Mcllwain tissue chopper (Ted Pella; Redding, California). The CA3 region was left intact for LTP but removed via a surgical cut in LTD experiments. Slices were transferred to a holding chamber with room temperature ACSF and allowed to recover for at least 1 h. On the day of experiment, the Aβ peptide/DMSO mixture was diluted to 100 μM in D-PBS, vortexed briefly and allowed to aggregate for 2 h at room temperature to form oAβ. The oAβ stock was then diluted to a final concentration of 500 nM in oxygenated ACSF and acutely applied to hippocampal slices for 2 h at room temperature. As an inclusion criterion to account for batch-to-batch variability, we routinely assessed the bioactivity of our oAβ preparation by confirming the ability of each oAβ batch to impair hippocampal LTP (Figures S1A and S1B).

#### Electrophysiology

After vehicle or oAβ incubation, a dorsal hippocampal slice was submerged in a Slicemate recording chamber (Scientifica; Uckfield, U.K.) with ACSF perfusing at a rate of 2.5 mL/min and temperature maintained at 30.0 ± 0.5°C. Extracellular field potentials were recorded from the stratum radiatum area of CA1 using a glass microelectrode (∼2-3 MΩ) filled with ACSF. Responses were evoked by stimulating the Schaffer collaterals using a platinum-iridium bipolar electrode (FHC; Bowdoin, Maine) positioned at the border between area CA2/CA1. Stimuli of 100 μs in duration were delivered once every 15 s via a DS2 constant voltage isolator (Digitimer; Hertfordshire, U.K.). Responses were amplified using a Multiclamp 700B amplifier (Molecular Devices; San Jose, California), digitized at 40 kHz and monitored in real time using WinLTP software (WinLTP Ltd.; Bristol, U.K).^103^ After a period of stable baseline recordings, datapoints for input/output (I/O) plots were obtained with fixed stimulus intensity at 1x, 1.5x, 2x, 3x, and 4.5x the threshold for evoking a visually detectable field excitatory postsynaptic potential (fEPSP) (≤ 0.05 mV). For plasticity experiments a stimulus intensity of 3x the threshold intensity was employed. LTP was induced by a theta-burst stimulation (TBS) protocol, with four stimuli delivered at 100 Hz, repeated 10 times at a frequency of 5 Hz. LTD was chemically induced by bath-application of (S)-3,5-DHPG (50 μM; Abcam; Cambridge, U.K.; cat. no. ab120007) for 10 min. In all experiments using receptor antagonists, antagonists were bath applied for a minimum of 30 min prior to the beginning of the baseline period until the end of the experiment. For offline analysis, responses were low-pass filtered at 2 kHz and the mean of four consecutive responses was quantified.

### Quantification and statistical analysis

Data in the time course plots were normalized to the mean of the entire baseline period (defined as 100 %) and presented as mean ± standard error of mean (SEM). The magnitude of LTP or LTD was quantified and presented as the percentage change of the fEPSP slope within the last 5 min of the recording *versus* the baseline period. Fiber volleys (FVs) and fEPSPs were quantified using peak amplitude and initial slope measurements, respectively, using WinLTP. The I/O plots were analysed using Microsoft Excel linear regression software. The respective slope values from a fitted line between FV *versus* stimulation intensity and fEPSP slope *versus* FV were used for quantification. Statistical comparisons between -oAβ and +oAβ conditions were assessed using an unpaired two-tailed Student’s t-test. The level of significance was set at p < 0.05. In all bar graphs, ∗ p < 0.05, ∗∗ p < 0.01, ∗∗∗ p < 0.001.
