# Supplementary material for: Amyloid-β1-42 oligomers enhance mGlu5R-dependent synaptic weakening via NMDAR activation and complement C5aR1 signaling
Source: iScience. 2023 Nov 7;26(12):108412. doi: 10.1016/j.isci.2023.108412 (PMC10694656; doi:10.1016/j.isci.2023.108412)
Supplement: Document S1. Figure S1 [file mmc1.pdf]

iScience, Volume *26*

Supplemental information

Amyloid-$\beta_{1\text{-}42}$ oligomers enhance $mGlu_5R$-dependent synaptic weakening via NMDAR activation and complement C5aR1 signaling

Ai Na Ng, Eric W. Salter, John Georgiou, Zuner A. Bortolotto, and Graham L. Collingridge

Supplemental Figure 1

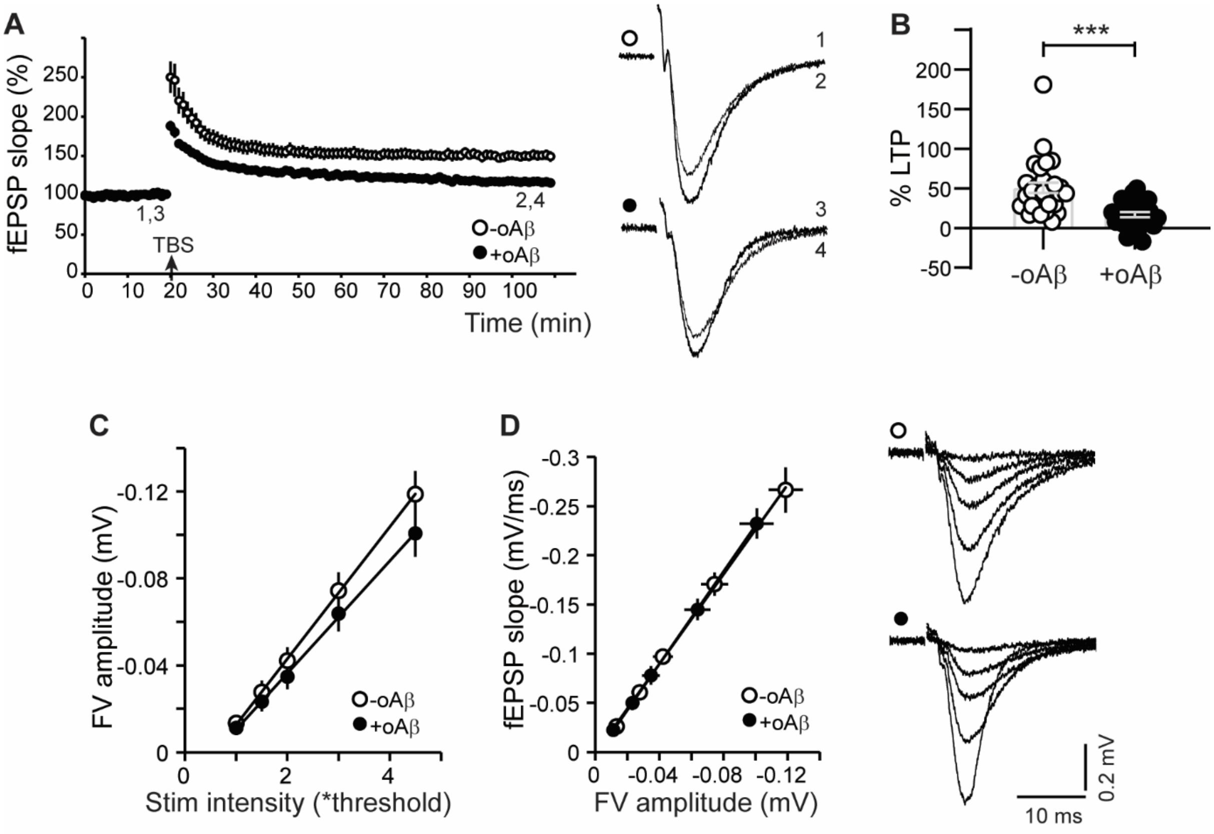

<u>Supplemental Figure 1: Acute oAβ application paradigm impairs LTP without affecting basal synaptic strength at CA1 synapses, related to Figure 1.</u>

(**A**, **B**) Induction of LTP by delivery of theta-burst stimulation (TBS), at the time indicated by the arrow. Magnitude of LTP is quantified in **B**. LTP magnitude was significantly lower in +oAβ slices (17 ± 3 %, n = 28) compared to interleaved -oAβ controls (51 ± 7 %, n = 28; p = 0.00002 [***]).

(**C**, **D**) Basal synaptic transmission strength was not significantly altered by acute oAβ application. **C** Fiber volley (FV) amplitude *versus* stimulus intensity relationship was not significantly different between treatment conditions (linear regression slopes: +oAβ = -

0.026 ± 0.003, n = 13; -oAβ = -0.030 ± 0.003, n = 13). **D** Input (FV amplitude) *versus* output (fEPSP slope) relationship was not significantly different between -oAβ and +oAβ conditions (linear regression slopes: +oAβ = 2.517 ± 0.237, n = 13; black, *versus* -oAβ = 2.513 ± 0.362, n = 13; white). *Right* Representative fEPSP traces at different stimulus intensity (multiples of threshold) for both conditions. In all graphs, data is presented as mean ± SEM.
